# MicroRNA-513b-5p inhibits epithelial mesenchymal transition of colon cancer stem cells through IL-6/STAT3 signaling pathway

**DOI:** 10.1007/s12672-024-01137-3

**Published:** 2024-07-05

**Authors:** Zefeng Zhang, Weihong Sha

**Affiliations:** Department of Gastroenterology and Digestive Endoscopy Center, Guangdong Provincial People’s Hospital (Guangdong Academy of Medical Sciences), Southern Medical University, Guangzhou, 510080 Guangdong China

**Keywords:** miR-513b-5p, IL-6/STAT3, Colon cancer stem cells, EMT, Metastasis

## Abstract

**Objective:**

To reveal the mechanisms by which miR-513b-5p inhibits metastasis of colon cancer stem cells (CCSCs) through IL-6/STAT3 in HCT116 cells.

**Methods:**

Sphere formation media and magnetic cell sorting were used to enrich and screen CCSCs. We used a colony formation assay, cell proliferation and viability assays, and a nude mouse transplantation tumor assay to identify CCSCs. ELISA was performed to identify IL-6 in the cell culture medium, and the growth, viability, wound healing, and transwell migration of distinct cell groups were compared to differentiate them. Dual-luciferase reporter assay, RT-PCR, and/or Western Blot analysis were conducted to determine the correlation between them.

**Results:**

CD133^+^CD44^+^ HCT116 cells were shown to have higher cloning efficiency, greater proliferation ability and viability, and stronger tumorigenicity. A dual-luciferase reporter assay revealed that miR-513b-5p negatively affected STAT3 expression. RT-PCR and/or Western Blot analysis suggested that miR-513b-5p negatively affected STAT3 and Vimentin, while positively affecting E-cadherin expression. The STAT3 overexpression vector + miR-513b-5p inhibitor cell group had the highest efficiency, greatest proliferation ability and viability, and the highest IL-6 level in the experiments.

**Conclusions:**

Mir-513b-5p inhibited the epithelial-mesenchymal transition (EMT) of CCSCs through IL-6/STAT3. This potential mechanism may provide a new therapeutic target for colon cancer.

## Introduction

Worldwide, an estimated 19.3 million new cancer cases and almost 10.0 million cancer deaths occurred in 2020 [1]. Colorectal cancer is the third most common cancer (10.0%) after breast cancer and lung cancer in women, and the second most common cause (9.4%) of death after lung cancer. Over 555,500 new cases were diagnosed, and nearly 286,200 deaths were recorded in China, accounting for nearly 30% of the cases in the world [1]. It has been reported that 85% of colorectal cancer patients are terminal when diagnosed, and the 5-year survival rate after surgery and chemotherapy is less than 40% [2]. The 5-year survival rate for colorectal cancer in China is far lower than that in developed countries such as Japan, South Korea, America, and Europe [3]. The metastasis and treatment of colon cancer remain important challenges for clinicians and researchers in China.

Cancer stem cells (CSCs) are a subpopulation of cancer cells with features including cancer recurrence, metastasis, and resistance to radiotherapy and chemotherapy [4–6]. Based on the theory of CSCs markers, it contributes to the screening, identification, and treatment of many human cancers, such as blood, head and neck, thoracic, abdominal, and genital system cancers [7–15]. Markers, such as CD133 and CD44, were found to be simultaneously expressed in colon cancer stem cells (CCSCs) [11–13] and related hepatic metastases [16]. Epithelial cells usually lose epithelial characteristics and acquire mesenchymal appearance or characteristics during cancer progression, which is defined as epithelial-mesenchymal transition (EMT) [17, 18]. To better understand the process, downregulation or loss of E-cadherin and upregulation of vimentin are regarded as markers of EMT [19].

MicroRNAs (miRNAs) are also involved in several physiological processes under normal conditions. They participate in various pathophysiological processes by regulating target genes via synergistic or inhibitory effects [20]. MiR-513b-5p was reported to be weakly expressed and related to the migration and invasion of colon cancer [21]. Signal transducers and activators of transcription 3(STAT3) are highly expressed and associated with colon cancer metastasis and prognosis [22] and play an essential role in maintaining the expression of the stem cell phenotype. Interleukin-6 (IL-6) is a cytokine secreted by a variety of cells that has extensive functions and multiple effects. IL-6 is often elevated in patients with colorectal cancer, especially in those with advanced cancer. Also, IL-6/STAT3 signaling pathway was reported to play a major role in the progression of gastric cancers [23, 24].

Our previous reports have revealed that the expression level of STAT3 in colon cancer tissues is highly associated with the invasion, migration, and progression of tumors. Here, we repeatedly screened and identified CCSCs in HCT116 cells, based on our previous research methods [25]. We also investigated the relationship between miR-513b-5p and STAT3 expression in CCSCs. This potential mechanism may provide a new therapeutic target for colon cancer.

## Materials and methods

### Cell line and cell culture

The HCT116 (GDCD625) colon cancer cell line, originating from the Chinese Academy of Science’s (CCTCC) cell bank, was collected for cell culture. RPMI1640 (Corning) supplemented with 10% fetal bovine serum (FBS) (Gibco) was prepared at 37 °C. Gbico culture with 5% CO2 tumor spheres of HCT116 cells were formed in suspension, as described in previous literature [26–28]. Composed of RPMI1640 (Peprotech), 20 ng/ml epidermal growth factor (Peprotech), 20 ng/ml fibroblast growth factor-basic (Peprotech), B27 (Gbico, 1:50 dilution), and 0 with Invitrogen (a bovine serum albumin, 4% in concentration), the Sphere Formation Media (SFM) was created. SFM was gradually substituted after the cells had been diluted to a concentration of 2 × 10^4^/ml, then cultured in 24-well plates. Each assay was carried out in triplicate.

### Flow cytometry

HCT116/SFM cells were resuspended in phosphate-buffered saline PBS (Corning) at a density of 1 × 10^7^/100 μl after washed twice with PBS. Cells After dissolved were stained with PE anti-human CD133 (1:10 dilution, Miltenyi Biotee) (cells after magnetic cell sorting (MACS) were not needed) and anti-human/Mouse CD44 (1:50 dilution, eBioscience), incubated for 20 min on ice, and washed twice with PBS. Simultaneously, the manufacturer’s instruction book (eBioscience) was employed to use the same concentration of respective isotype controls. A flow cytometer (FACS-verse, BD Biosciences) was used to analyze the samples. Each assay was carried out in triplicate.

### Magnetic cell sorting

HCT116/SFM cells were resuspended in PBS with 2% FBS at a density of 1 × 10^8^/ml to a total volume of 1 ml, using a 12 × 75 mm polystyrene tube to make it suitable for the magnet (EasySep) 0.100 μl anti-human CD32 (Fcγ RII) blocker, 50 μl of PE anti-human CD133, 100 μl of PE selection cocktail and 50 μl of magnetic nanoparticles were sequentially added based on the manufacturer’s directions (EasySep). The cell suspension was augmented with PBS and 2% FBS, resulting in a total volume of 2.5 ml (Step A). Subsequently, the magnet was filled in the tube for 5 min, after which the supernatant fraction was poured out (Step B). Steps A and B are repeated twice. Magnetically labeled cells were kept within the tube, relying on the magnetic field of the magnet. Flow cytometry and other experiments necessitated the capture of cells in tubes. Each assay was carried out in triplicate.

### Colony formation assay

In each 6-well plate, 50,100,200 CD133^+^CD44^+^ HCT116 and 50, 100, 200 CD133^−^CD44^−^ HCT116 cells were seeded. The medium was poured off when colonies could be observed by the naked eye. Methanol and 10% Giemsa were used to fix and stain colonies. Each assay was carried out in triplicate.

### Cell counting kit-8 viability and proliferation assays

Cells were cultured for approximately 4 h until they attained complete adherence, after which 96-well plates were placed with 100 μl of suspension (CD133^+^CD44^+^ HCT116 cells, CD133^−^CD44^−^ HCT116 cells, and RPMI1640 solution). Cells were seeded at a population of 1000, 2000, 4000 and 8000 cells per well. After 10 μl of 2-(2-methoxyl-4-nitrophenyl)-3-(4-nitrophenyl)-5-(2,4-two sulfonatophenyl)-2H-tetrazolium monosodium salt (Cell Counting Kit-8(CCK8) (Dojindo) was added to each well, and the plate was incubated for approximately 4 h. A spectrophotometer (Multiskan GO, Thermo) was used to determine the absorption (OD value) at 450 nm. The OD values were corrected after subtracting that of RPMI1640, as a control. Each assay was carried out in triplicate.

### Transplantation tumor assay in BALB/C-nu/nu mice

CD133^−^CD44^−^ HCT116 and CD133^+^CD44^+^ HCT116 cells were trypsinized, pelleted, and resuspended in RPMI1640 with Matrigel (BD, 1:10 dilution). BALB/C-nu/nu mice were randomly divided into seven groups and the breeder assigned to the project was randomly assigned to six groups. 200 μl of cell suspension (2000, 20,000, 200,000 CD133^+^CD44^+^ cells or 2000, 20,000, 200 cells) was then used.000 Subcutaneously, BALB/C-nu/nu mice were injected with either a CD133^−^CD44^−^ or NaCl solution. The tumor size and body mass of the mice were determined at regular intervals. The maximal tumor size was required to be less than 2000 mm^3^ (Volume = 1/2ab^2^, a = Long axis of the tumor; b = short axis of the tumor) during the breeding by our ethics committee. After 4 weeks, the tumors were euthanized by cervical dislocation following an intravenous injection of pentobarbital sodium (100 mg/kg). This study conformed to the ARRIVE 2.0 guidelines [29] and followed the guidelines [30].

### Plasmid construction and transfection of STAT3

The target and mutant sequences of the STAT3 plasmid were as: TATCATGCCGATGAAGCATG and GGCAGCAAATGTTCGTAGGT. The control sequence was custom-ordered and offered by XFL Medical Biotechnology Co., Ltd. (Guangzhou, China). CD133^+^CD44^+^ HCT116 cells were diluted to 2 × 10^3^/well and transfected using Lipofectamine 2000 (Invitrogen, USA) according to the manufacturer’s instructions.

### Dual-luciferase reporter assay

Plates of 24 wells were filled with 2 × 10^4^ cells per well and cultured in DMEM containing a high glucose concentration. OPTI-MEM (Invitrogen) was supplemented with 300 μL, 1 μL lipofectamine 2000 (Invitrogen), 50 nM microRNA or 100 nM microRNA inhibitor, and 0.5 μg plasmid. The procedure was gradually augmented with 5 μg of plasmid. Utilizing the Dual-Luciferase Reporter Assay System (E1910, Promega) to assess luciferase activity in specimens, the manufacturer’s instructions were followed. The has-513b-5p and its control (normal) and has-513b-5p inhibitor and its control (normal inhibitor) were provided by XFL Biotechnology Co., Ltd (Guangzhou, China). Each assay was carried out in triplicate.

### IL-6 in cell culture medium detected by ELISA assay

Cell culture medium was diluted with sample diluents after centrifugation to remove precipitates and was plated at 100 μL/well in 96-well plates. Then, was added 100 μL biotin labelled anti human IL-6 antibody,100 μL ABC working fluid, and 90 μL TMB substrate and 100 μL stop solution were added according to the manufacturer’s instructions (ZN2272, Baiaolaibo). The absorbance (OD value) was measured at 450 nm using a spectrophotometer (Multiskan GO, Thermo). Each assay was carried out in triplicate.

### Real-time PCR

Total RNA from cultured cells was extracted with Trizol Reagent (TaKaRa), and 1.0 μg of this was then used for cDNA synthesis with PrimeScript RT reagent Master Mix (TaKaRa). Using the SYBR Premix ExTaq II (TLiRNaseH Plus) (TaKaRa) and the ABI PRISM 7500 Fast Real-Time PCR System (Bio-Rad), real-time PCR was performed in the following order: 95 °C for 30 s, 95 °C for 5 s, 60 °C for 1 min, and 95 °C for 30 s, for 40 cycles. The 2^−∆∆CT^ technique was employed to further examine the results, with β-actin gene expression functioning as an endogenous control. Each assay was carried out in triplicate.

### Western blot analysis

Lysate buffer (150 mM NaCl, 0.5% sodium deoxycholate, 0.1% NP40, and 50 mM Tris pH 7.4) was used to lyse cells, and the BSA method was then employed to measure the protein concentration of the lysate. SDS–polyacrylamide gels, which were then transferred onto nitrocellulose membranes, were used to load and separate lysates. To impede the membranes, 5% nonfat milk powder in TBS was administered for 1 h. Primary antibodies against STAT3(D3Z2G) rabbit mAb (#12640,1:1000 dilution, Cell Signalling Technology, CST) and vimentin (D21H3) rabbit mAb (#5741,1:1000 dilution, Cell Signalling Technology, CST) were then used to probe the membranes. The Mouse mAbs (#14472, 1:1000 dilution, Cell Signalling Technology, CST) of E-cadherin (4A2) and GAPDH (KC-5G4, 1:8000 dilution, Kangchen, Shanghai) were both tested. After washing with TBS-T, the membranes were incubated with secondary antibodies (A21020, HRP Goat anti-rabbit; A21010, HRP Goat anti-mouse, 1:6000 dilution, Abbkine). Chemiluminescence in the Image Quant LAS 500(GE) was used for visualization. Each assay was carried out in triplicate.

### Wound healing assay

Six-well plates were used to culture 5 × 10^5^ cells per well until they reached a point of convergence. A PBS triple wash was used to create a diametric scratch with the aid of a pipette tip. Cells in several pre-marked spots were photographed at 0 h as controls. Photographs of the same spots were obtained at 48 h for comparison. Imagepro plus 6.0 (Media Cybernetics) was used to measure the scratch width and compare migration rates of each group of cells on average. Each assay was carried out in triplicate.

### Transwell migration assay

A concentration of 300 μg/ml of Matrigel matrix (BD) was formulated, and 24-well plates were covered with 100 μl per well. After incubation for 1 h, RPMI1640 was used to resuspend the cells at a concentration of 1 × 10^5^/ml. After 48 h of incubation at 37 °C with 5% CO2, 500 μl of the RPMI1640, containing 20% FBS was placed in the lower chamber and 100 μl of cell suspension in the upper chamber. The filter was fixed with ethanol, and 10% Giemsa stain was applied. A microscope was employed to calculate the number of cells migrating to the underside of the membrane, and a cotton swab was used to purify the cells on the upper side of the filter. Nine microscopic fields (100 × magnification) were used to randomly select cells. Each assay was carried out in triplicate.

### Statistical methods

Data of the means and SEMs are presented. Variance analysis and Student’s t-test were used to determine the statistical significance of discrepancies between groups. Statistical significance was set at P < 0.05.

## Results

### CCSCs were enriched, screened and identified in vivo and vitro

**Fig. 1:**
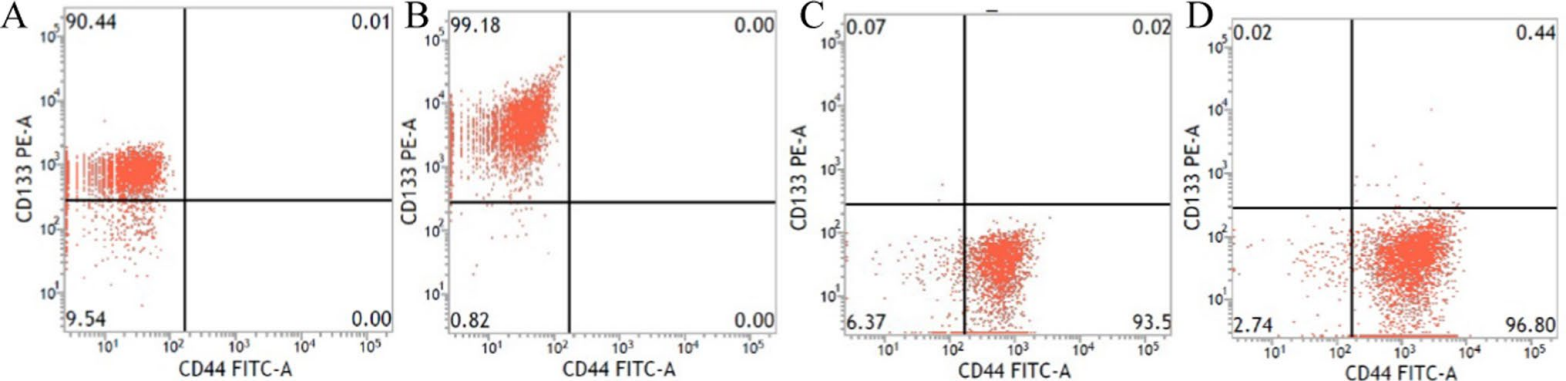
CD133 and CD44 propottion of HCT116 cells analyzed by flow cytometry. **A**, **B** The proportion of CD133^+^ HCT116 cells after SFM and MACS were from 90.44 to 99.18%; **C**, **D** the proportion of CD44^+^ of CD133^+^ HCT116 cells after SFM and MACS were from 93.5 to 96.8%

Before SFM and MACS, the CD133^+^CD44^+^ subpopulation of the cultured HCT116 cells accounted for less than 1.00% and were regarded as CD133^−^CD44^−^ cells. CD133^+^CD44^+^ HCT116 cells were significantly improved after SFM culture and MACS in our experiment, and they were identified as CCSCs. Notably, the proportion of CD133^+^CD44^+^ HCT116 cells was largely determined by CD133^+^ HCT116 cells. The proportion of CD133^+^CD44^+^ cells after SFM and MACS was presented as (92.91 ± 2.60) % analyzed by flow cytometry (Fig. 1).

**Fig. 2:**
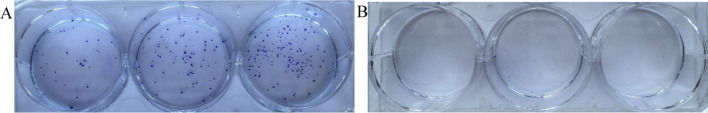
Colonies of cells after stained with 10% Giemsa by naked eyes. **A** cloning efficiency of CD133^+^CD44^+^cells was (88.44 ± 5.54) % on average; **B** cloning efficiency of CD133^−^CD44^−^cells was (8.12 ± 2.68) % on average. The difference between the two groups was significant, P < 0.05. Each assay was carried out in triplicate

Colony formation assay was performed to detect the self-renewal and differentiation abilities of CSCs. The cloning efficiency of CD133^+^CD44^+^ HCT116 cells was significantly higher than that of CD133^−^CD44^−^ HCT116 cells (Fig. 2 and Table 1) after staining with 10% Giemsa with the naked eye.

**Table 1:** Comparison of cloning efficiency of the two group cells

Groups	CD133^+^CD44^+^ HCT116 cells	CD133^−^CD44^−^ HCT116 cells	Difference
Cloning efficiency	(88.44 ± 5.54) %	(8.12 ± 2.68) %	P < 0.05

Cell Counting Kit-8 (CCK8) was used to further validate CSC function through cell proliferation and viability assays. The experimental results and manufacturer’s instructions revealed a distinct difference between the 8000 cells of each group, with OD values of (1.08 ± 0.07) and (0.74 ± 0.08) (P < 0.05). Then, another 8000 cells of each group were cultured as previously described to compare cell viability, which was repeated for 30 wells. The values of 8000 cells in each group on average were (1.00 ± 0.12) and (0.83 ± 0.11) (P < 0.05).

**Fig. 3:**
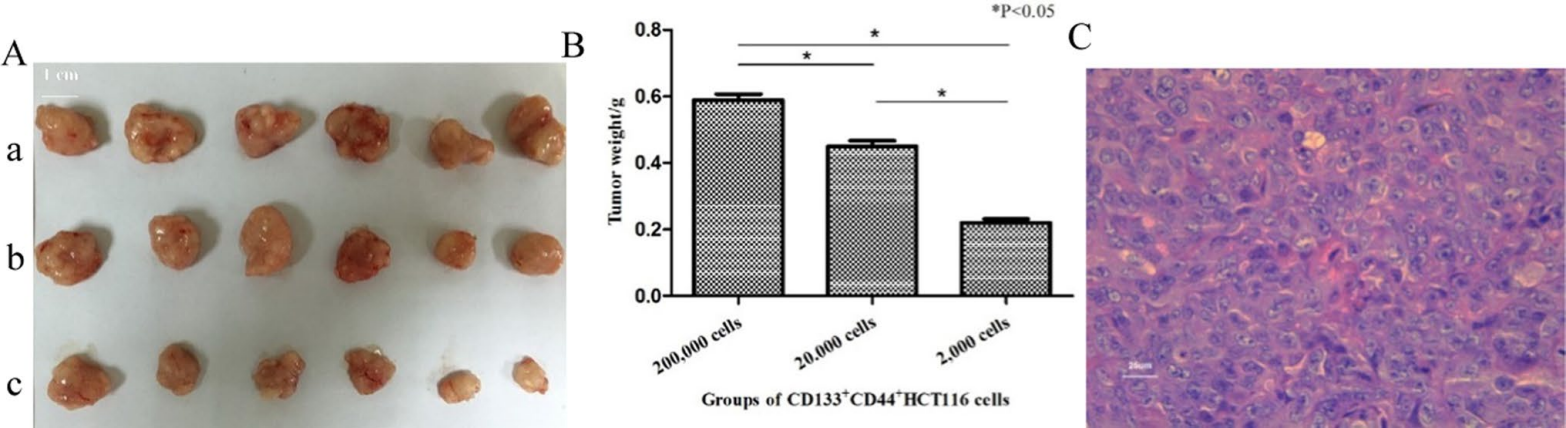
Transplantation tumor assay in BALB/C-nu/nu mice. **A**-**a** tumor masses of 200,000 CD133^+^CD44^+^group; **A**-**b** tumor masses of 20,000 CD133^+^CD44^+^group; **A**–**C** tumor masses of 2,000 CD133^+^CD44^+^group; **B** tumor weights of **A**/**B**/**C** groups were (0.59 ± 0.07) g, (0.45 ± 0.13) g and (0.22 ± 0.16) g, respectively; P < 0.05. **C** routine pathological section examination

A transplantation tumor assay was performed to confirm that CCSCs have strong tumorigenicity in vivo. BALB/C-nu/nu mice were fed for 4 weeks after the solution injection. Only the CD133^+^CD44^+^group (100%) developed tumors after 4 weeks, which were also concentration-dependent (Fig. 3A). All the volume of tumor were less than 2000 mm^3^. The tumor weights of the three groups of CD133^+^CD44^+^ cells were significantly different (P < 0.05, Fig. 3B). All BALB/C-nu/nu mice were sacrificed to remove tumors and for routine pathological examination (Fig. 3C) to ensure that the assay was successful.

### Mir-513b-5p impacted negatively on STAT3 and IL-6

**Fig. 4:**
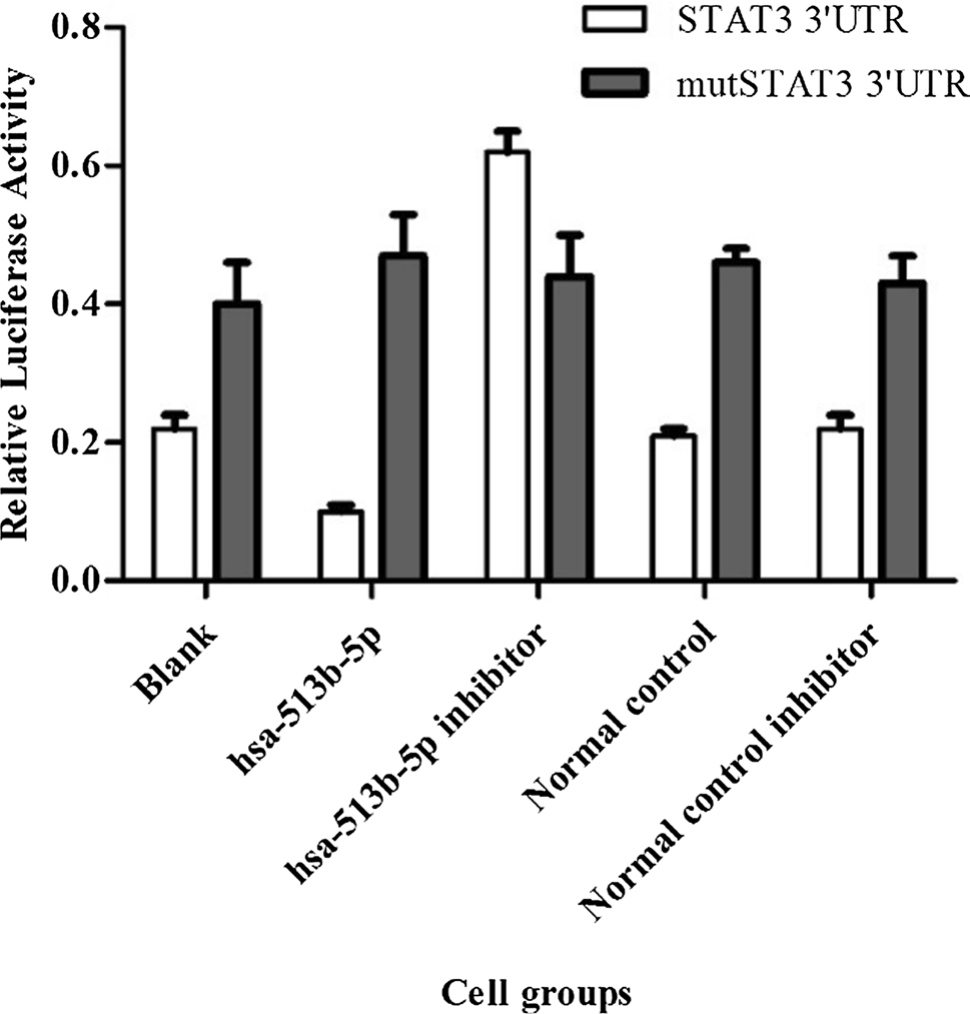
Relative luciferase activity of STAT3 in Dual-Luciferase Sensor Reporter Group has-513b-5p (0.10 ± 0.01) and has-513b-5p inhibitor (0.62 ± 0.03) were significantly different from the other 3 groups (0.22 ± 0.02), P < 0.05. (Normal control: control of has-513b-5p, normal control inhibitor: control of has-513b-5p inhibitor) Each assay was carried out in triplicate

**Fig. 5:**
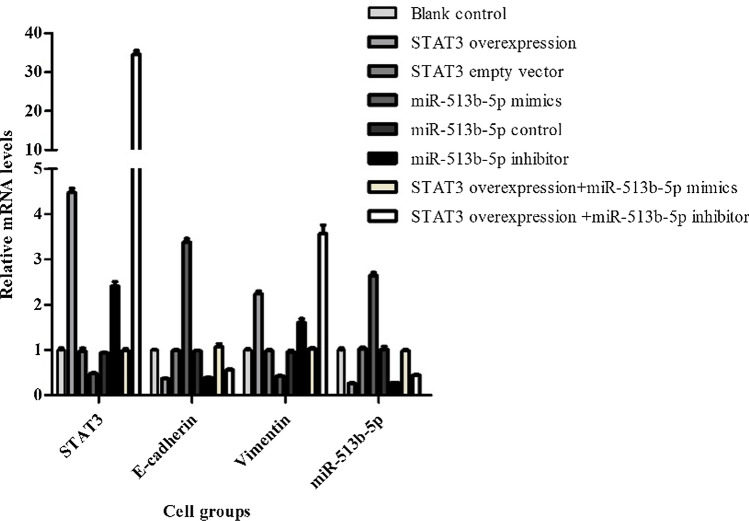
Relative mRNA expression of STAT3, E-cadherin, Vimentin and miR-513b-5p in different cell groups. **A** blank control group; **B** STAT3 overexpression vector group; **C** STAT3 empty vector group; **D** miR-513b-5p mimics group; **E** miR-513b-5p control group; **F** miR-513b-5p inhibitor group; **G**. STAT3 overexpression vector + miR-513b-5p mimics group; **H**. STAT3 overexpression vector + miR-513b-5p inhibitor group. Group **B** and **D**/**G**/**H**, Group **D** and **F**, Group **G** and **H**, P < 0.05. Each assay was carried out in triplicate

**Fig. 6:**
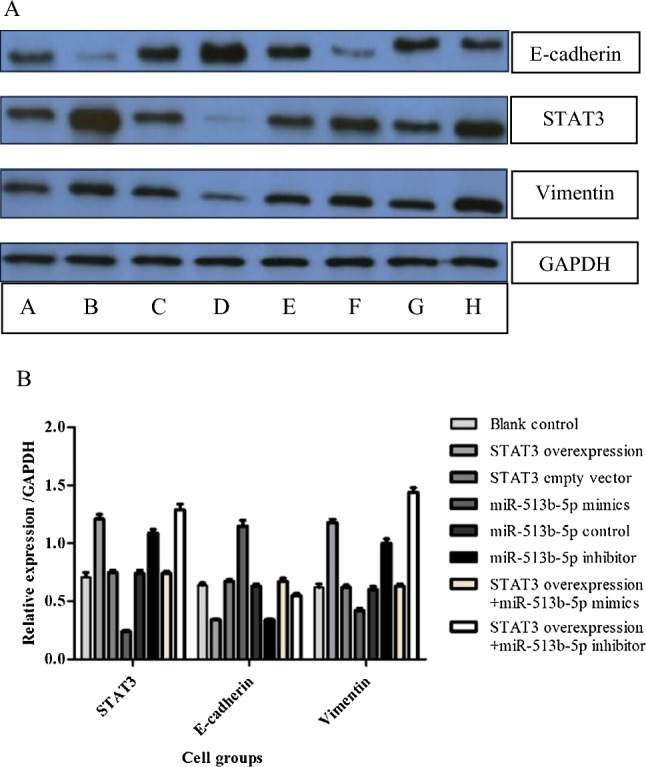
Expression of protein STAT3, E-cadherin, Vimentin and GAPDH (Western blot) in different cell groups group. **A** blank group; group **B** STAT3 overexpression vector group; group **C** STAT3 empty vector group; group **D** miR-513b-5p mimics group; group **E** miR-513b-5p control group; group **F** miR-513b-5p inhibitor group; group **G** STAT3 overexpression vector + miR-513b-5p mimics group; group **H** STAT3 overexpression vector + miR-513b-5p inhibitor group. STAT3: group **B** with **A**/**C**/**D**/**E**/**G**; E-cadherin and Vimentin: group **B** with **A**/**C**/**D**/**E**/**G**/**H**; P < 0.05. Each assay was carried out in triplicate

A dual-luciferase reporter assay was conducted to assess the relationship between miR-513b-5p and STAT3. The relative luciferase activity of STAT3 in has-513b-5p group was much lower and in the has-513b-5p inhibitor group was much higher than that in the blank group (Fig. 4). Meanwhile, there was no difference among the mut-STAT3 3′ UTR groups as a control. In addition, from the PCR and Western blot results, among the different 8 groups, we also found that miR-513b-5p negatively affected STAT3 expression (P < 0.05, Figs. 5, 6).

**Fig. 7:**
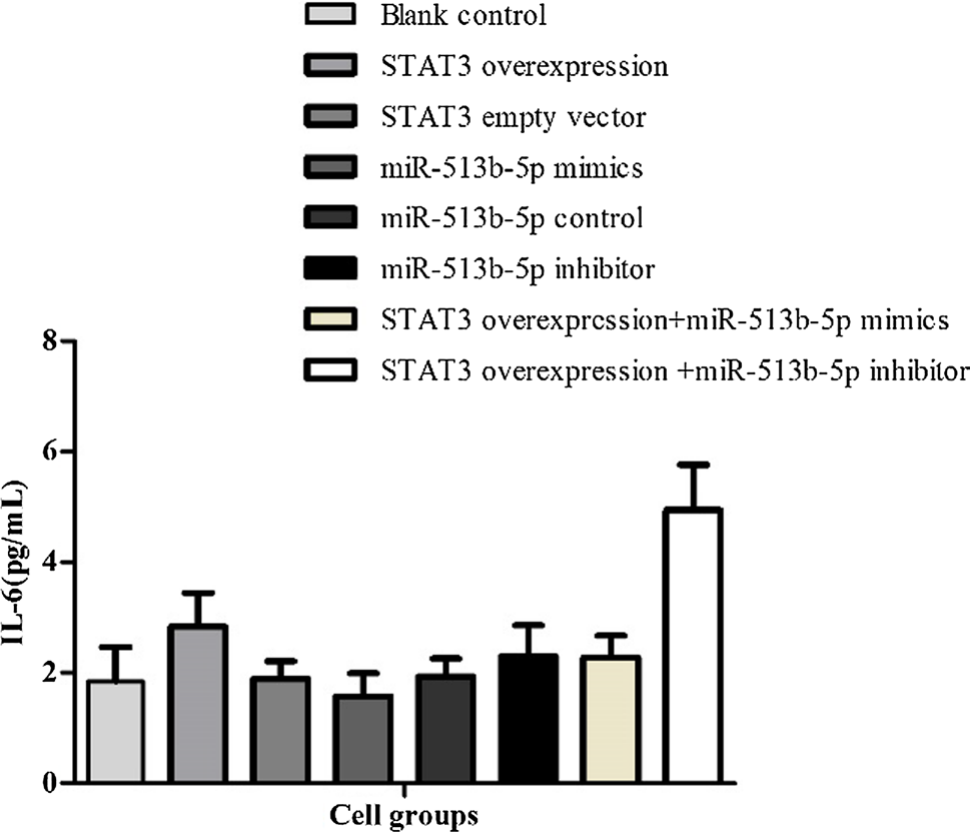
Level of IL-6 in cell supernatant of different groups. STAT3 overexpression vector group as (2.83 ± 0.61) pg/mL, miR-513b-5p inhibitor group as (2.30 ± 0.56) pg/mL and STAT3 overexpression vector + miR-513b-5p inhibitor group as (4.94 ± 0.82) pg/mL were much higher than the miR-513b-5p mimics group as (1.57 ± 0.42) pg/mL and the controls, P < 0.05. Each assay was carried out in triplicate

In addition, we found a positive correlation between IL-6 and STAT3 levels. IL-6 in cell culture medium in STAT3 overexpression vector group as (2.83 ± 0.61) pg/mL, miR-513b-5p inhibitor group as (2.30 ± 0.56) pg/mL and STAT3 overexpression vector + miR-513b-5p inhibitor group as (4.94 ± 0.82) pg/mL were much higher than the miR-513b-5p mimics group as (1.57 ± 0.42) pg/mL and the controls (P < 0.05, Fig. 7).

### Mir-513b-5p inhibits EMT of CCSCs through IL-6/STAT3

**Fig. 8:**
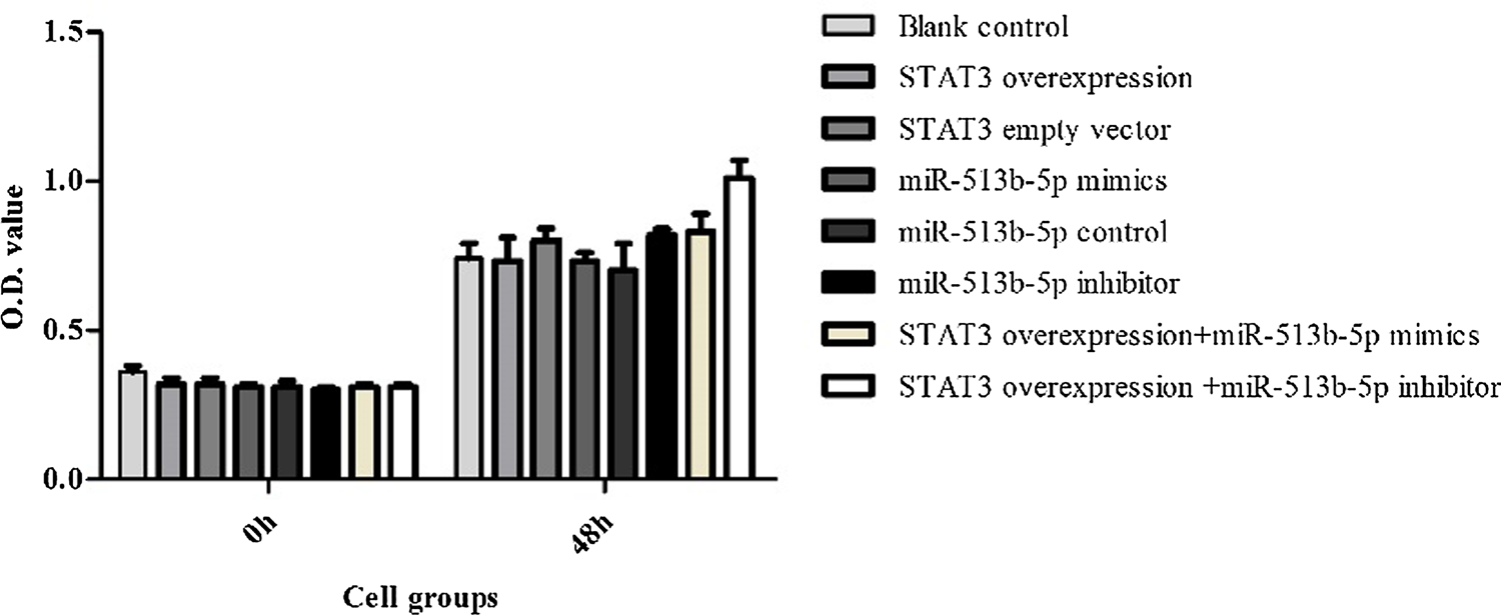
Viability (OD. value) of different cell groups cells using the CCK-8 Blank control (**A**) group 48 h: 0.74 ± 0.05; STAT3 overexpression vector (**B**) group 48 h: 0.73 ± 0.08; STAT3 empty vector (**C**) group 48 h: 0.80 ± 0.04; miR-513b-5p mimics (**D**) group 48 h: 0.73 ± 0.02; miR-513b-5p control (**E**) group 48 h: 0.70 ± 0.09; miR-513b-5p inhibitor (**F**) group 48 h: 0.82 ± 0.02; STAT3 overexpression vector + miR-513b-5p mimics (**G**) group 48 h: 0.83 ± 0.06; STAT3 overexpression vector + miR-513b-5p inhibitor (**H**) group 48 h: 1.01 ± 0.06. Group **H** and **B**/**F**/**G**, Group **F** and **D**/**E**, P < 0.05. Each assay was carried out in triplicate

**Fig. 9:**
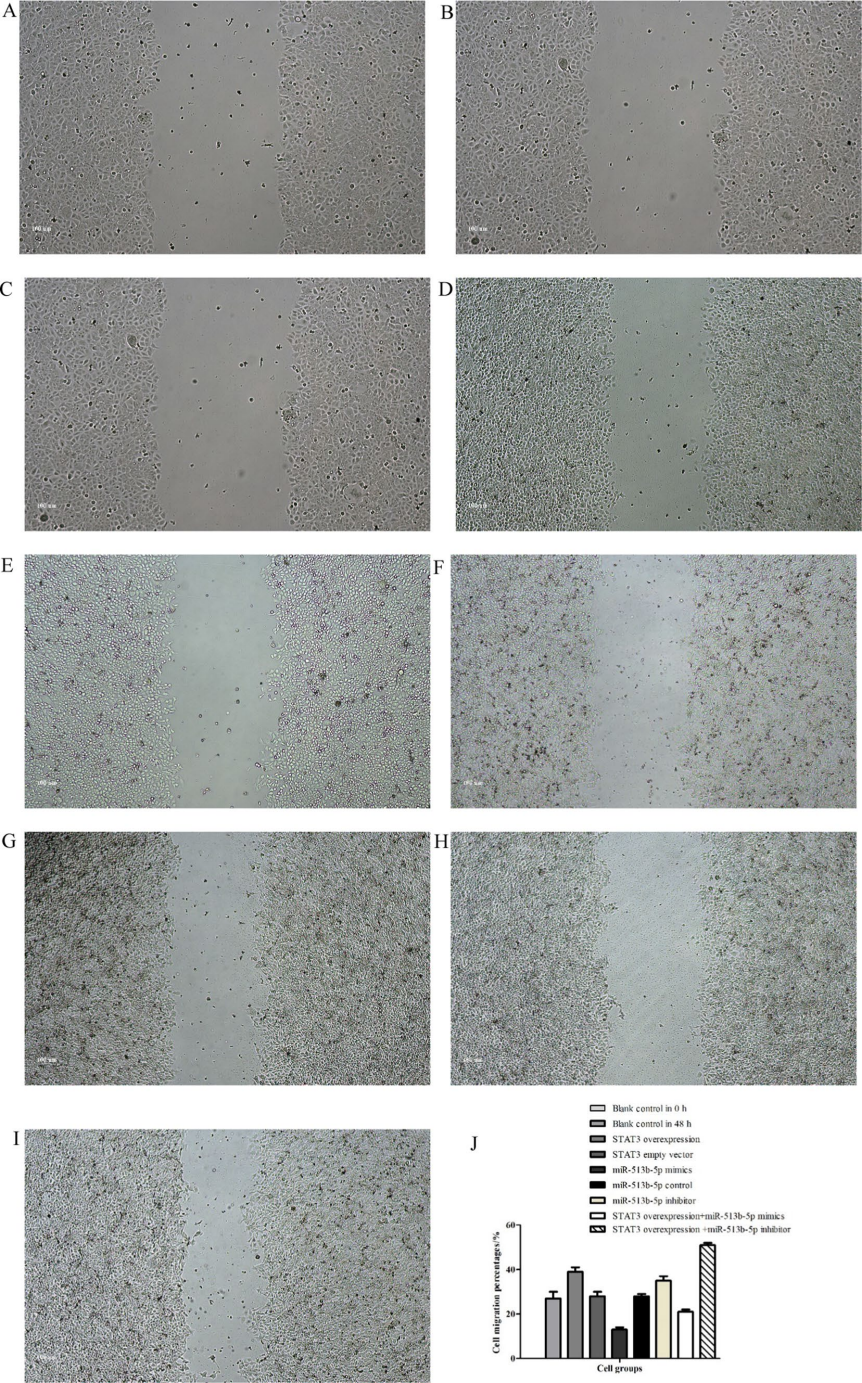
Wound healing (cell migration percentages) of CCSCs seeded in 6-well plates and cultured after 48 h. **A** blank control group in 0 h; **B** blank control group in 48 h: (27.00 ± 3.00)%; **C** STAT3 overexpression vector group: (39.00 ± 2.00)%; **D** STAT3 empty vector group: (28.00 ± 2.00)%; **E** miR-513b-5p mimics group: (13.00 ± 1.00)%; **F** miR-513b-5p control group: (28.00 ± 1.00)%; **G** miR-513b-5p inhibitor group: (35.00 ± 2.00)%; **H** STAT3 overexpression vector + miR-513b-5p mimics group: (21.00 ± 1.00)%; **I** STAT3 overexpression vector + miR-513b-5p inhibitor group: (51.00 ± 1.00)%. **J** Group **B** and **C**/**E**/**G**/**H**/**I**, Group **E** and **G**, Group **H** and **I**, Group **C** and **H**/**I**, P < 0.05. Each assay was carried out in triplicate

**Fig. 10:**
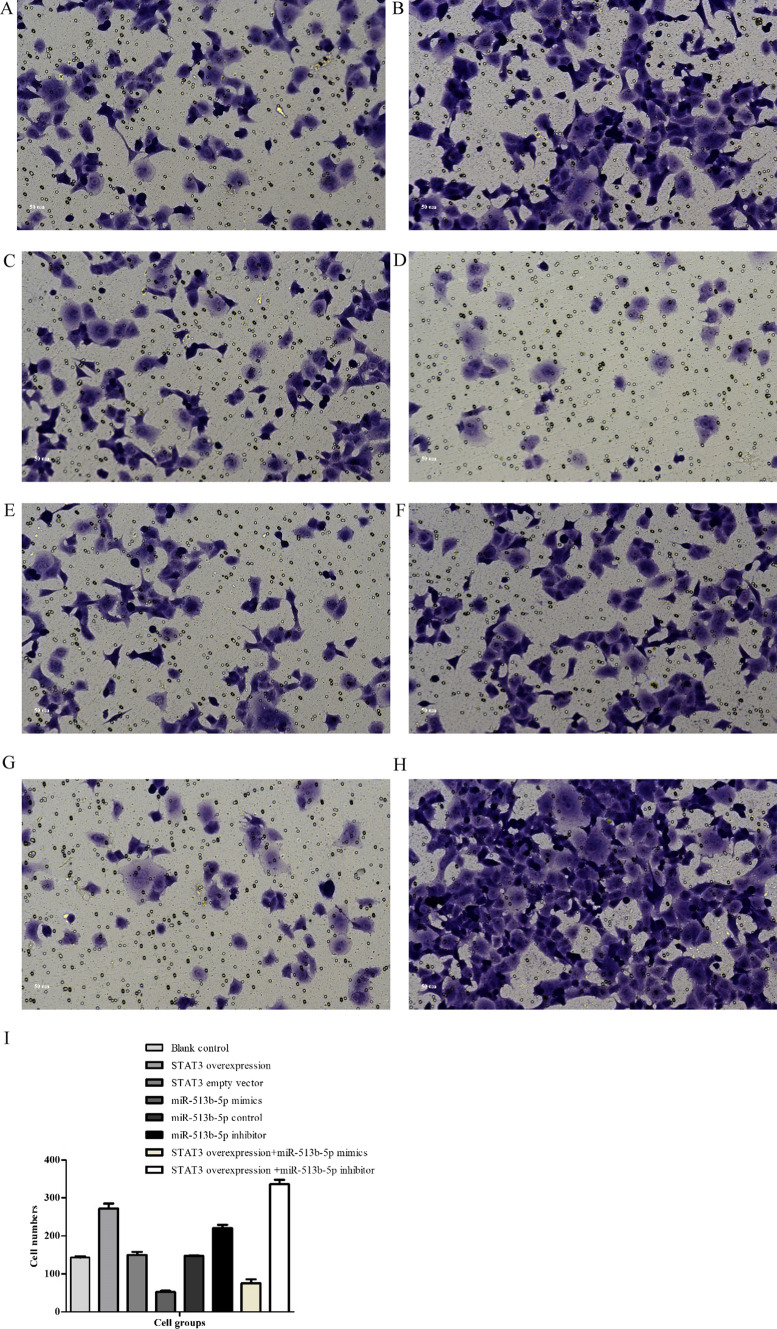
Cell numbers in the undersurface of Transwell membrane after 48 h. **A** blank group: 143.33 ± 3.21; **B** STAT3 overexpression vector group:272.00 ± 13.11; **C** STAT3 empty vector group:149.67 ± 8.50; **D** miR-513b-5p mimics group:52.33 ± 3.21; **E** miR-513b-5p control group:147.33 ± 1.53; **F** miR-513b-5p inhibitor group:220.67 ± 8.50; **G**. STAT3 overexpression vector + miR-513b-5p mimics group:75.00 ± 10.58; **H** STAT3 overexpression vector + miR-513b-5p inhibitor group:336.00 ± 11.79; all were observed under a microscope (200 ×) after stained with 10% Giemsa. **I** Group **A** and **B**/**D**/**F**/**G**/**H**, Group **D** and **F**, Group **G** and **H**, Group **B** and **G**/**H**, P < 0.05. Each assay was carried out in triplicate

Together with the above results of PCR and Western blot (Figs. 5, 6 and 7), we also found miR-513b-5p impacted positively with E-cadherin, while it impacted negatively with STAT3 and Vimentin. We further performed CCK8, would healing and transwell assays to identify cell functional changes among different groups. STAT3 overexpression vector + miR-513b-5p inhibitor group had more cell viability and proliferation ability (1.01 ± 0.06, Fig. 8), higher migration percentages [(51.00 ± 1.00) %, Fig. 9] and more invaded cell numbers (336.00 ± 11.79, Fig. 10) than other groups in our study. It seemed that miR-513b-5p inhibited EMT of CCSCs through IL-6/STAT3 signaling pathway.

## Discussion

Colorectal cancer is the first and second most common cause of gastrointestinal cancer in China in recent years [1]. In China, 85% of colorectal patients are diagnosed with advanced cancer, and the 5-year survival rate after surgery and chemotherapy is less than 40%. In contrast, developed countries such as Europe, America, Japan, and South Korea have remarkably high 5 year survival rate [3]. The incidence and metastasis of colorectal cancer are problems that cannot be neglected in the West and East, especially in China. Studies on the prevention of the progression and metastasis of colon cancer should be a priority for researchers in this field.

Isolation and acquisition of CSCs is the first step in their application in basic and clinical medicine. Both sphere formation media (SFM) and magnetic cell sorting (MACS) have an advantage in the enrichment and screening of CSCs. In vitro and in vivo functional experiments are typically performed to assess CSCs. Our research revealed that CD133^+^CD44^+^ HCT116 cells could be employed as CCSCs in further studies [25]. Based on our results, we concluded that CD133^+^CD44^+^ HCT116 cells were CCSCs.

Markers such as CD133 and CD44, both early discovered as vital biomarkers of CCSCs [8–10], are recommended to screen and identify CCSCs together. We speculate that different cell markers may represent different functions and help fully understand the overall features. To better understand this, CD133 may be related to cloning efficiency and proliferation. CD44 may be associated with metastasis and survival [13]. Membrane proteins such as CD24 [31], CD26 [32, 33], CD29 [34], CD166 [35], Lgr5 [36–38] and EpCAM [39, 40]; cytosolic enzyme ALDH1 [41, 42]; transcription factors such as Ascl2 [43–45], Oct4 [46], Sox2 [47], and Hes1 [48, 49]; and Wnt [50] and Notch [51] signaling pathways have all been reported as markers of CCSCs. It was also recently reported that recombinant doublecortin-like kinase 1 (DCLK1) can be considered a marker of CCSCs [52].

The discovery and eradication of CSCs have a bright future in cancer treatment; the same is true for genes applied to CSCs and related regulatory mechanisms. Signal transducers and activators of transcription 3 (STAT3) are highly expressed and associated with colon cancer metastasis and prognosis [22] and play an essential role in maintaining the expression of the stem cell phenotype. STAT3 is regarded as a promising target for colitis-associated cancers [53]. Interleukin-6 (IL-6) is a cytokine with extensive functions and multiple effects, and is secreted by a variety of cells. The IL-6/STAT3 signaling pathway plays a major role in the progression of gastric cancers [23, 24]. This suggests that STAT3 is a positive regulator of colon cancer progression, and downregulation of STAT3 may contribute to the prevention of invasion and migration of colon cancer.

To date, over 1400 miRNAs have been identified in humans. It has been reported that 30–60% of protein-coding genes are regulated by miRNAs, although they only make up 1–3% of the human genome [54]. miRNAs may serve as a new approach for stem cell research. They participate in various pathophysiological processes by regulating target genes via synergistic or inhibitory effects [20]. In previous studies, miR-513b-5p was shown to inhibit proliferation and induce apoptosis in retinoblastoma [55], hepatocellular carcinoma [56], ovarian cancer [57], and pancreatic cancer [58]. MiR-513b-5p was also reported to be weakly expressed and related to the migration and invasion of colon cancer, including the HCT116, RKO, SW480, LoVo and SW620 cell lines [21], and SW116 cell line [59], and colon cancer tissues [60]. Meanwhile, miR-1299 was found to be a negative regulator of ST AT3 in colon cancer [61], which targeted deletion of miR-139-5p, activating STAT3 signaling and promoting progression of colorectal cancer [62], and miR-125b inhibited cell proliferation by targeting STAT3 [63]. However, to date, a relationship between miR-513b-5p and IL-6/STAT3 in CCSCs has not been reported. In our study, we demonstrated that miR-513b-5p was expressed at low levels in CCSCs and negatively affected IL-6/STAT3. It was also shown that miR-513b-5p inhibited CCSCs invasion and migration through IL-6/STAT3.

The transmembrane protein E-cadherin inhibits tumorigenesis and metastasis of tumors, and the downregulation or loss of E-cadherin results in the shedding of different types of cancer cells from the tumor mass; this process is called EMT [64, 65]. Along with the interaction between miR-513b-5p and STAT3, we also found that miR-513b-5p positively affected E-cadherin, while it negatively affected vimentin. Furthermore, the corresponding cell groups showed improved proliferative, invasive, and migratory capacities in our experiments. This observation suggests that miR-513b-5p inhibits the EMT of colon CCSCs through IL-6/STAT3.

microRNA helps to predict potential drug resistance or acquire drug resistance, and to synthetize analogues or inhibitors by targeting specific targets; which are combined with anti-cancer drugs to regulate the expression of major proteins in drug metabolism, thereby affecting personalized cancer treatments. In colon cancer drug-resistant cell line LoVo/5-Fu, mir-210 was significantly upregulated, while let-7fmir-1228 was significantly downregulated [66]. It was speculated that the miR-1915 regulating BCL-2 apoptotic pathway to reduce oxaliplatin resistance in HCT116/L-OHP [67]. MiR-145 was also reported could reduce oxaliplatin resistance in human colon cancer HCT116 cell line by affecting the expression levels of MDR1 and PTEN genes and proteins [68]. These measures will offer new targets for treatment and improve the clinical therapy effects of colon cancer. However, there still are many challenges before the clinical applications. The stability and effectiveness of mir-513b-5p are still needed to overcome in the future. Nano-delivery technology such as nanoparticles seems to be a promising technique.

Here are some limitations. We performed the study mainly in the CCSCs model in HCT116 cell line, not in all colon cancer cell lines and did not reveal reducing drug resistance mechanisms. It is worth mentioning that the role of mir-513b-5p in colon cancer is obvious and effective in cell experiment, but the stability and effectiveness of mir-513b-5p are still needed to overcome and the specific anti-cancer drug increasing therapeutic efficacy should be elaborated before the clinical applications.

It is important to put forward the idea of miR-513b-5p, EMT, and IL-6/STAT3 in CCSCs. In summary, mir-513b-5p inhibits EMT of CCSCs through IL-6/STAT3 pathway. This potential mechanism may provide a new therapeutic target for colon cancer.

## Conclusion

Taken together, our results indicate that CD133^+^CD44^+^ HCT116 cells can be used as CCSCs in further medical research. miR-513b-5p inhibits the EMT of CCSCs through IL-6 and STAT3. This potential mechanism may provide a new therapeutic target for colon cancer.

## Data Availability

The data that support the findings of this study are available from the Department of Gastroenterology, Guangdong Provincial People’s Hospital; however, restrictions apply to the availability of these data, which were used under license for the current study, and so are not publicly available. However, data are available from the corresponding author upon reasonable request and with permission from Gastroenterology, Guangdong Provincial People’s Hospital.
